# Therapeutic Potential of Brazilian Cerrado *Campomanesia* Species on Metabolic Dysfunctions

**DOI:** 10.3390/molecules23092336

**Published:** 2018-09-13

**Authors:** Carla Maiara Lopes Cardozo, Aline Carla Inada, Gabriela Marcelino, Priscila Silva Figueiredo, Daniela Granja Arakaki, Priscila Aiko Hiane, Claudia Andrea Lima Cardoso, Rita de Cássia Avellaneda Guimarães, Karine de Cássia Freitas

**Affiliations:** 1 Post Graduate Program in Health and Development in the Central-West Region of Brazil, Federal University of Mato Grosso do Sul-UFMS, Campo Grande 79070-900, MS, Brazil; carlinhalopescardozo@hotmail.com (C.M.L.C.); inadaaline@gmail.com (A.C.I.); gabi19ac@gmail.com (G.M.); pri.figueiredo92@gmail.com (P.S.F.); daniarakaki@gmail.com (D.G.A.); priscila.hiane@ufms.br (P.A.H.); ritaaguimaraes@gmail.com (R.d.C.A.G.); 2 Research Center in Biodiversity, Faculty of Chemistry, State University of Mato Grosso do Sul, Dourados 79804-970, MS, Brazil; claudia@uems.br

**Keywords:** *Campomanesia* species, medicinal plants, Brazilian Cerrado, obesity-induced metabolic syndrome

## Abstract

Obesity, in conjunction with other metabolic disorders such as insulin resistance and dyslipidemia, is a feature of metabolic syndrome which is characterized by a pro-inflammatory state and increased oxidative stress. Therefore, antioxidant foods are proposed to prevent and treat these disorders. Medicinal plants are one of the main strategies in this regard. Guavira, a Brazilian Cerrado plant, contains different bioactive compounds with a high antioxidant capacity and without clinical or reproductive toxicity effects. Though there are different varieties of guavira, the principal Brazilian Cerrado guaviras demonstrated hypoglycemic, anti-inflammatory, and hypocholesterolemic actions. There is also a potential antiplatelet agent in *C. xanthocarpa*, while *C. adamantium* displayed hypocholesterolemic actions in animal models and human clinical trials. On the other hand, even with a lack of studies related to *C. pubescens*, it demonstrated anti-inflammatory effects and an antioxidant capacity in in vitro studies. Despite the fact that most of the studies were not performed to evaluate pathological conditions specific to obese animal models or obese subjects, guavira demonstrated effects in metabolic disorders that are commonly related to the obesity context, such as cardiovascular disturbances and hyperglycemia status. This suggests that guavira is a potential therapeutic approach to obesity-induced metabolic syndrome.

## 1. Introduction

### 1.1. Obesity-Induced Metabolic Syndrome and Perspectives in Medicinal Plants

Obesity is a condition that involves a set of metabolic disorders and is characterized by an energy unbalance in which there is a high energetic uptake with lower energetic expenditure. It is a well-known risk factor for the development of chronic diseases which are related to the individual′s lifestyle [1]. It has a strong correlation with type 2 diabetes (DM2), in which obese individuals are at risk patients to develop DM2 and glucose intolerance [2]. These are often seen together with dyslipidemia which is more observed in obese patients than non-obese subjects [3]. In this way, the hyperglycemic profile in obese patients is associated to acute insulin resistance which is similar to metabolic syndrome (MetS) which, in turn, is characterized by insulin resistance, hypertension, central obesity (abdominal fat), and dyslipidemia. Therefore, patients with MetS display a prothrombotic and pro-inflammatory state that elevates the risk of developing stroke, coronary heart disease, peripheral vascular disease, and DM2, leading to a high incidence of mortality rate from cardiovascular diseases (CVDs) [4].

Obesity-induced metabolic syndrome is predominantly a result of the synthetic function of adipose tissue due to the fact that this tissue displays endocrine and paracrine functions through the activity of cytokines and chemokines, which are known as adipokines [5]. The increased adipose tissue mass causes a state of metabolic inflammation with high production of pro-inflammatory mediators, such as tumor necrosis factor (TNF-α), interleukins (e.g., IL-6, IL-8, IL-1β), and angiotensin II, which is correlated to hypertension. There are also decreased levels of anti-inflammatory cytokines such as adiponectin, another adipokine with an important role in glucose management, vasculo-protective effects, anti-inflammatory, and anti-atherogenic properties [5,6,7,8].

In this way, this pro-inflammatory profile on obesity-induced metabolic syndrome is a key factor in the stages of atherosclerosis, such as in the progression and destabilization that precedes myocardial infarction, and also in the induction of a hypercoagulable state leading to an increase in fibrinogen and plasminogen activator inhibitor, which inhibits fibrinolysis [2]. In addition to these factors that leads to the evolution of atherosclerosis, the abnormal lipid profile characterized by high levels of serum triglycerides (TG), an increase in serum lipoproteins, such as very low density lipoprotein (VLDL-c) and also in low density lipoprotein (LDL-c), and a reduction in high density lipoprotein (HDL-c) are common in diabetic obese-induced metabolic syndrome individuals [6,7].

Moreover, another deleterious factor which is increased in MetS and DM2 and seems to underlie the progress of CVDs is oxidative stress, and this condition appears to lead to insulin resistance, dyslipidemia, pancreatic β-cell dysfunctions, impaired glucose tolerance, and, consequently, DM2 [9]. Oxidative stress is a condition that is characterized by an imbalance between free radicals and the antioxidant defense mechanisms. Therefore, the increased reactive oxygen species (ROS) may result in degradation of lipids, proteins, and nucleic acids and, thereby, result in oxidative cell damage. This, in turn, is suggested to play a major role in pathogenesis of diseases, causing increased risks of insulin resistance, dyslipidemia, elevated blood pressure, metabolic syndrome, inflammation, and endothelial dysfunction [10]. It is established that ROS levels are increased in obesity, especially in central (abdominal) obesity, being the major component of MetS [11]. In addition, others studies have demonstrated that augmented oxidative stress is associated with insulin resistance and adipokines dysregulation [12,13].

For this reason, the maintenance of redox homeostasis possesses an important action in the prevention of diseases and health support [10,14]. These metabolic dysfunctions relating to obesity-induced metabolic syndrome may be significantly decreased by dietary modifications, physical activity, and antioxidant drugs. These are therapeutic approaches targeting oxidative stress, preventing or delaying the progression and onset of diseases [9,14]. Another alternative, as a source of therapeutic approach, is the use of traditional medicines or medicinal plants. Some edible fruits and vegetables are a rich source of antioxidants. It was observed that antioxidants show health benefits, reducing the oxidative stress through different mechanisms, such as ROS scavenging, chelating metals, and terminating lipid peroxidation [10].

In general, even with a large amount of knowledge on antioxidant structures, biological actions, and properties in the literature, there is still a lack of scientific basis for the use of medicinal plants in this practice since little is known about appropriate dosages, effectiveness of treatment, cellular mechanisms, and interactions between the bioactive compounds which are necessary clarifications. Despite the fact that research on medicinal plants′ therapeutic potential on obesity-induced metabolic syndrome has increased substantially, specific medicinal plants in different biomes are important to study due to the fact that there is an immense potential of natural products that are still unknown that potentially have novel therapeutic approaches in several metabolic diseases.

### 1.2. Medicinal Plants: The Potential of Brazilian Cerrado

Medicinal plants are widely used in folk medicine and are counted as traditional medicines since they have played a primary role in health for thousands of years. These plants have been used in the treatment of various health problems and this vast consumption has been in the form of powder, gums, teas, oils, or associated forms [15]. The use of medicinal plants as an alternative or additional therapeutic resource has increased significantly [16]. This is because even through synthetic drugs are usually the first choice of treatment for many diseases, these chemical drugs sometimes have undesirable effect. Consequently, the acceptance of alternative medicines has grown substantially [17].

The World Health Organization (WHO) estimates that one-third of the world′s population, such as in some parts of Latin America, Africa, and Asia, have no access to essential regular remedies; due to this, the rich natural resources of traditional medicines are the first choice for this population due to their availability and affordability [18]. Thus, natural-based products have become increasingly popular, which explains the interest in research of this field [17].

The importance of the study and use of medicinal plants is due to the presence of bioactive compounds that are responsible for their beneficial effects on health, increasing the curiosity of researchers to understand these effects since these components may act in a synergistic form or a specific compound may be responsible for the principle effects. In general, there are many types of compounds, including alkaloids, terpenoids, flavonoids, pigments, tannins, and many others [19]. In several countries around the world, such as China, India, and Africa, medicinal plants continue to be a therapeutic complement, or even the basis, for the treatment of diseases [20].

South America is considered the territory with one of the greatest biodiversity in terms of fruit and plant species, with the Amazon as the main Brazilian biome in terms of biodiversity. Another important biome is the Brazilian Cerrado, which is the second largest biome, comprising 21% of the land area [21]. It is located in the middle west of Brazil, possessing a vast diversity of native fruits and plants. Even with some studies displaying their properties on health, such as antioxidant properties [22], most of these are still unknown. However, Brazilian Cerrado edible fruits have a great potential for agricultural use and they are extensively used by the locals due to popular accessibility, together with economic contribution, since these plants are used as local income for their producers and are also important food options [21,23]. These fruits are manually collected and are consumed raw or in the form of food-products, such as juice, liquor, ice-cream, and various types of sweets [21].

Studies about plants from the Brazilian Cerrado has increased considerably. These plants include graviola (*Annona muricata* Linnaeus), which has displayed anti-ulcerogenic effects [24], bocaiuva (*Acrocomia aculeata*) which has presented anti-diabetic properties [25] and anti-inflammatory and diuretic actions [26], and baru (*Dipteryxalata* Vog.), which was observed to cause improvement in lipid profiles and atherogenic indexes [27]. Another important plant from the Brazilian Cerrado is pequi (*Caryocar brasiliense*) which has displayed anti-inflammatory and antioxidant properties, decreasing hepatic damage induced by carbon tetrachloride in rats [28]. Among other species used with medicinal purposes are the genus *Campomanesia*, belonging to the family Myrtaceae, which is popularly known as guavira [21]. It has demonstrated effects on health, such as antiulcerogenic [29], antiprotozoal [30], anti-inflammatory [31,32], antioxidant [33,34,35,36,37], and antiproliferative [38,39] activities.

### 1.3. Campomanesia (Myrtaceae)

The Myrtaceae family possesses approximately 133 genii and more than 4000 species around the world, with the *Campomanesia* genus one of them [40]. The genus *Campomanesia* possesses around 30 species. Of these, 24 species may be found in Brazil [41] and is popularly known as guavira, guabirobeira, or guabiroba, characterized by fruits with a citrus aroma and flavor. Moreover, the fruit may be used as fresh products in the food industry due to its attributes, such as a considerable amount of ascorbic acid acidity, mineral, fiber, and monoterpene hydrocarbons [21].

There are several varieties of *Campomanesia*, including *Campomanesia xanthocarpa*, *Campomanesia adamantium*, *Campomanesia corymbosa*, *Campomanesia cambessedeana*, *Campomanesia pubescens*, and many other species [22].

Besides the fruits being used as potential food products, some studies have demonstrated the effects of this plant on health. Previous studies have demonstrated anti-ulcerogenic effects from *C. xanthocarpa* [29] and anti-inflammatory activity [31,32]. Due to these positive effects on health and because of the concerns that involve metabolic dysfunctions associated to obesity-induced metabolic syndrome, a current study was conducted which demonstrated hypocholesterolemic, antioxidant, and anti-inflammatory effects in humans from *C. xanthocarpa* [42] and hypocholesterolemic effects in rodent models from *C. adamantium* [37].

Considering the advances in the studies of medicinal plants and their bioactive compounds, notably in prevention and/or treatment of metabolic dysfunctions, and given the recent and potential discoveries with the varieties *C. xanthocarpa*, *C. adamantium*, and *C. pubescens*, the aim of this study is to gather the most important studies related to the Brazilian *Campomanesia* species and determine the therapeutic potential of the common Brazilian Cerrado *Campomanesia* species in metabolic dysfunctions which may or may not be associated to obesity-associated metabolic syndrome, focusing on the possible mechanism of action in in vivo, including animal models and human clinical trials, and in vitro assays.

## 2. Common Brazilian Cerrado *Campomanesia* Species as a Potential Therapy for Metabolic Dysfunctions

### 2.1. Campomanesia xanthocarpa O. Berg

*C. xanthocarpa* O. Berg is found in the midwest [22] and southern region of Brazil, as well as in Argentina, Paraguay, and Uruguay [43]. Some studies have demonstrated its important therapeutic activities, such as antiulcerogenic [29], anti-inflammatory [32], and antiprotozoal [30] effects, as well as antidiarrheal and antimicrobial activity [44], antioxidant potential [33], and antiplatelet, antithrombotic, fibrinolytic [45,46], and, more recently, hypotensive effects [47] from *C. xanthocarpa*.

These therapeutic activities are due to the presence of bioactive compounds demonstrated in most of the studies. An important study has showed that fruits from guavira display a high quantity of phenolic compounds including chlorogenic acid (9033 mg/100 g) and vitamin C (30.58 mg/g). Moreover, its antioxidant activity, which was evaluated through the 2,2-azino-bis-3-ethylbenzothiazoline-6-sulfonic (ABTS) method, exhibited the highest antioxidant potential (507.49 µM Trolox/g) in comparison to other fruits like uvaia (*Eugenia pyriformis* Cambess.) and yellow guava (*Psidium cattleyanum* Sabine) [33].

The phytochemical profile of both leaf and fruit hydroalcoholic extracts of guavira revealed the presence of flavonoids, saponins, and tannins [29,44], which were associated to the antiulcerogenic effect of the hydroalcoholic extract of guavira leaves in female Wistar rats [29]. Other effects from fruit hydroalcoholic extract, such as an antimicrobial effect in bacterial test strains and antidiarrheal activity, were analyzed by testing intestinal motility in female mice [44].

Apart from anti-inflammatory effects and pain treatment, recently novel bioactive compounds were identified in extracts of leaves. These components 2′,6′-dihydroxy-3′-methyl-4′metoxychalcone and 2′,4′-dihydroxy-3′,5′-dimethyl-6′-methoxychalcone, could inhibit paw edema induced by carrageenan injection and diminish leucocyte migration into the pleural cavity in adult male Wistar rats [32]. In addition to these beneficial effects on health, toxicity evaluation is an important parameter to be analyzed on studies of edible plants. The extract of leaves of *C. xanthocarpa* and isolated compounds have not displayed clinical signals of toxicity [29,32] or reproductive toxicity [48].

In the face of the increasing prevalence of metabolic disturbances related to obesity, some studies assessed the potential use of *C. xanthocarpa* considering the variables of hypercholesterolemia and hyperglycemia, which are two conditions associated to obesity and related to the development of DM2 and cardiovascular events [6]. A preliminary study has evaluated the infusion of guavira leaf aqueous extract on weight control in male Wistar rats that received a high calorie diet totaling approximately 3.8 cal/g in 30 days. The guavira infusion group had significantly diminished weight gain concomitant with decreased blood glucose levels and a 15% reduction in plasma glucose level when compared to the group that received only a high calorie diet without treatment. Nonetheless, the hypocholesterolemic effect was not observed in these animals [49].

Despite the hypocholesterolemic profile not being observed, the hypoglycemic action corroborates the previous study after a three-week treatment with *C. xanthocarpa* leaf decoction (20 g/L), which may be useful for DM management in streptozotocin-induced (STZ, 70 mg/kg/body weight) diabetic Wistar rats without a significant variation on serum lipids. On the condition of STZ treatment, these induced diabetic rats are considered a typical model of DM1 and the drug was administered one week prior to the treatment with leaf decoction, causing typical diabetes symptoms, such as polydipsia, polyuria, polyphagia, hyperglycemia, hypertriglyceridemia, and histopathological modifications in the liver, kidney, and pancreas [50].

Only the rats with a blood glucose above 13.875 mmol/L (250 mg/dL) were considered diabetic. Therefore, the findings have demonstrated that STZ-treated rats which were treated with leaf decoction have exhibited decreased blood glucose levels (26% reduction) in comparison to the STZ-treated rats which received water [50]. In fact, other pharmacological studies related to medicinal plants with hypoglycemic properties consider 12% to 20% reduction in blood glucose levels a significant reduction [51]. Moreover, STZ-induced diabetic rats treated with *C. xanthocarpa* displayed inhibited hepatic glycogen loss, suggesting that the treatment could restore or preserve liver glycogen. According to the histopathological analysis of the pancreas and kidneys, the *C. xanthocarpa* decoct was able to preserve the histopathological alterations in the pancreas without changes on the shape of islet and the presence of insulin was observed [50].

In addition, diabetic rats have presented typical diabetic nephropathy. However, the *C. xanthocarpa* decoct group did not display glomerular or tubular pathological alterations, suggesting that the treatment may reduce the damage in the pancreas and have an anti-inflammatory effect on the kidneys in STZ-induced diabetic rats [50]. Even though the phytochemicals profile was not evaluated in this study, one suggestion about the hypoglycemic effects promoted by *C. xanthocarpa* decoct was reported in a previous study which has demonstrated that the presence of flavonoids, such as quercetin, myricetin, quercitrin, and rutin, may be responsible for the effects of the decoction [52].

Besides hyperglycemia, hyperlipidemia is another condition that presents a key role in the development of the micro- and macrovascular complications of diabetes [53], together with cardiovascular events [6]. Due to popular usage of *C. xanthocarpa* in the prevention and treatment of CVD, some experimental studies have been carried out in in vivo and in vitro experimental models to verify the *C. xanthocarpa* effect as antiplatelet agent. Therefore, it is important to expose that the integration between platelets and blood vessels plays a fundamental role in the maintenance of cardiovascular integrity, in which uncontrolled platelet aggregation leads to arterial thrombosis [54,55]. In this way, antiplatelet agents possess an important tool in the prevention and the treatment of CVD, such as atherosclerosis. Acetylsalicylic acid (ASA) a common example of this antiplatelet agent used for many individuals [56]. However, even though ASA is an effective secondary drug to prevent ischemic cardiovascular disorders, it can produce some important side effects such as bleeding events, in conjunction with gastrointestinal bleeding [57].

In this way, a current study has evaluated the effects of *C. xanthocarpa* leaf extract in knockout mice for the low-density lipoprotein receptor (LDLr-KO), an experimental model that mimics hypercholesterolemia in humans displaying not only hypercholesterolemia, but also vascular inflammation. First of all, the groups were fed with a hypercholesterolemic diet for four weeks; thereafter, the animals were divided in different groups of treatment. The first group received distilled water, the second was treated with *C. xanthocarpa* leaf extract (100 mg/kg), and the last with ASA (100 mg/kg), an anti-inflammatory that inhibits platelet aggregation and inflammation in low doses, preventing cardiovascular mortality. Treatment totaled five days in all the groups [58].

Considering that inflammation is a common condition of atherosclerotic disease, *C. xanthocarpa* leaf extract was able to attenuate proinflammatory markers, such as IL-6, IL-1, TNF-α, and IFN-γ, and to increase IL-10 serum levels in LDLr-KO mice. This is different from the ASA mice group which has not displayed significant impacts on inflammatory markers, but showed an increase of a less percentage on IL-10 levels when compared with the *C. xanthocarpa* group. Furthermore, *C. xanthocarpa* and ASA could diminish serum levels of oxidized low-density lipoprotein (ox-LDL). However, only *C. xanthocarpa* was able to decrease anti-oxLDL antibodies without ulcerogenic activity, which is considered one of the important side effects promoted by ASA [58].

One condition that is associated with atherosclerosis is the accumulation of LDL-c in the intima-media layer of the artery, together with endothelial dysfunction. The latter, in turn, may result in structural modifications with the accumulation of LDL-c in the arterial intima and, consequently, lead to the formation of atheroma plaques [59,60]. Thereafter, this accumulation of LDL-c may be oxidized and lead to the formation of oxLDL, which results in an increase of adhesion molecules and the secretion of proinflammatory cytokines by endothelial cells with consequent infiltration of monocytes and macrophages within the intima. These immune cells release more cytokines, adhesion molecules, and chemokines that influence atherogenesis with the rupture of atherosclerotic erosions, leading to the local aggregation of platelets [61].

Thus, the reduction of ox-LDL promoted by *C. xanthocarpa*, together with the decreased formation of anti-oxLDL, showed that the plant displayed an antioxidant potential since anti-oxLDL may enhance the removal serum ox-LDL and, therefore, avoid ox-LDL entrance in the arterial wall [58]. Furthermore, the principle bioactive compounds from *C. xanthocarpa* that were responsible for these effects were gallic acid, chlorogenic acid, quercetin, and rutin [58], supporting previous studies that have demonstrated that flavonoids and phenolic acids are the main phytochemicals found in the same plant [62,63].

In accordance with this data, Klafke et al. [45] have evaluated antiplatelet, antithrombotic, and fibrinolytic activity in mice and in humans. Swiss male mice were treated orally for five days with *C. xanthocarpa* leaf extract in different doses (30 and 100 mg/kg/day) or ASA (100 mg/kg/day). Therefore, blood samples were collected from the hepatic vein of these animals to evaluate bleeding, acute thromboembolism, and ulcerogenic activity in addition to prothrombin time (PT) and activated partial thromboplastin time (aPTT). Moreover, mice and blood samples from healthy human participants were evaluatedvia anin vitro assayof the antiplatelet effects by artificial blood clot degradation. The effect of cytotoxicity of *C. xanthocarpa* on platelets were analyzed through the leakage of lactate dehydrogenase (LDH) from human platelets [45].

The *C. xanthocarpa* leaf extract was able to inhibit synthetic adenosine diphosphate (ADP) [45], a potential drug that induces the interaction of membrane receptor glycoprotein IIb–IIIa with fibrinogen, which performs platelet aggregation during thrombus formation. Besides that, *C. xanthocarpa* showed no cytotoxic effect, suggesting a natural source with antithrombotic effects due to the ability to inhibit platelet aggregation and through fibrinolytic activity [45].

Furthermore, the *C. xanthocarpa* extract could prolong aPTT without differences on the PTtest, suggesting a slightly anticoagulant effect measured by the aPTT test. Both the PT and aPTT tests are used in clinical tests of blood coagulation [45]. Thrombin participates crucially in many physiological conditions and pathological procedures, such as thrombosis, inflammation, blood coagulation, tumor growth, and metastasis [64,65,66]. Given the role of thrombin in the development of a thrombus, many strategies focus on inhibiting thrombin generation and blocking its activity, thus preventing and treating thromboembolic events [67].

In accordance with the conventionally used dose of ASA (100 mg/kg), *C. xanthocarpa* leaf extract displayed favorable gastric effects without presenting gastric lesions when compared to ASA, suggesting that the extract is safer than ASA [45]. These effects may be justified due the presence of saponins, tannins, terpenes, and a small presence of flavonoids [68].

The antiplatelet activity from *C. xanthocarpa* is in accordance with another study that used healthy subjects (*n* = 23) who were divided in three groups. One group was treated with ASA (100 mg), another was treated with *C. xanthocarpa* leaf extract (1000 mg), and the last group is a synergism group who was treated with both ASA (50 mg) and *C. xanthocarpa* leaf extract (500 mg) daily, totaling five days of treatment [46].

Antiplatelet activity was evaluated by the turbidimetric method using ADP and arachidonic acid (AA) agonists before five and eight days after treatment ended, respectively. The group that was treated with *C. xanthocarpa* (1000 mg) and the synergism group (*C. xanthocarpa* 500 mg + ASA 50 mg) exhibited a reduction in platelet aggregation induced by ADP. Using AA as an agonist, all groups obtained a decrease in platelet aggregation; however, *C. xanthocarpa* (1000 mg) and ASA (50 mg) groups have a shorter antiplatelet effect compared to the synergism group (*C. xanthocarpa* 500 mg + ASA 50 mg), which displayed a prolonged antiplatelet effect due to the maintenance of aggregation reduction eight days after the treatment ended. This suggests that this group′s treatment is the best therapeutic approach to influence platelet aggregation [46].

Considering that uncontrolled platelet aggregation and hypercholesterolemia are two important components to the development of CVD, a preliminary study was performed in adult subjects (*n* = 33) with undesirable levels (200–240 mg/dL) of total cholesterol (TC) and hypercholesterolemic individuals (>240 mg/dL) [68]. These subjects were divided randomly into a control group that was treated with placebo capsules and the others received either 250 or 500 mg of encapsulated *C. xanthocarpa* leaf powder extract for 90 days. The data did not show any difference in hematological profile (such as hematocrit and hemoglobin), weight and abdominal circumference, oxidative stress parameters (measured by protein carbonyl levels in plasma), and some biochemical parameters after the treatment with the extracts. However, a significant decrease was observed in TC and LDL-c levels in hypercholesterolemic patients which received encapsulated *C. xanthocarpa* leaf powder extract (500 mg) when compared with the control group [68].

In order to investigate whether the diminished levels of TC may be associated to the free radical scavenger activity of *C. xanthocarpa* leaf extract, anti-oxidant activity tested through the ABTS test together with a 3-hydroxy-3-methylglutaryl-CoA reductase (HMGR) activity assay were performed. HMGR is an enzyme which has an activation state influenced by the increase of reactive oxygen species (ROS) and is the key enzyme of cholesterol biosynthetic pathway [69,70,71]. The *C. xanthocarpa* leaf extract displayed a high anti-oxidant capacity and a concentration-dependent inhibitory activity on HMGR, probably due the inhibition of the catalytic subunit of the enzyme. This suggests that this mechanism works in parallel to the mechanism of HMGR inhibitors [68].

Moreover, the presence of some phytochemicals, like saponins, tannins, terpenes, and a small presence of flavonoids in *C. xanthocarpa* is suggested to cause the decrease of hypercholesterolemia, since bioactive compounds act as reactive oxygen species scavengers [68]. These results corroborate another important clinical study of the same group which has evaluated the influence of encapsulated leaves extract of guavira on adult subjects (between 18 and 90 years old). This study was conducted in a double-blind fashion and randomized, with patients (*n* = 156) who met the criteria for a hypercholesterolemia profile [42].

These hypercholesterolemic individuals have received different doses of encapsulated *C. xanthocarpa* extract: 500, 750, or 1000 mg per day. The control group received a placebo treatment, an encapsulated excipient (lactose), totalling three months of treatment. After the treatment, participants that received the 500 mg *C. xanthocarpa* leaf extract presented a decrease in LDL-c and total cholesterol levels when compared to the control group. There were no differences in triglyceride, VLDL-c, or HDL-c levels [42].

Beyond the lipid profile, other parameters such as high-sensitivity C-reactive protein (hs-CRP), high levels of which indicate an inflammatory process, and oxidative stress parameters, such as advanced oxidation protein products (AOPPs) and ischemia-modified albumin (IMA) which indicate an oxidative stress process at high levels, were evaluated. In addition, nitric oxide (NO), which plays a protective function by decreasing abnormal proliferation of vascular smooth cells and lower levels of which indicate endothelial dysfunction, was evaluated in the study. The data showed a significant reduction in oxidative stress and inflammatory components concomitant with a significant increase in NO in hypercholesterolemic subjects which were treated with encapsulated 1000 mg *C. xanthocarpa* leaf extract, when compared to the control group given a placebo. This suggests that *C. xanthocarpa* promotes the reduction of inflammation and oxidative stress, and displayed protective effects on the endothelium [42].

Another relevant pathological condition considered a cardiovascular risk factor for morbidity and mortality in the world is essential hypertension. A current study accomplished by Sant’Anna et al. [47] evaluated the influence of *C. xanthocarpa* leaf extract on systolic and diastolic blood pressure and heart rate. Normotensive Wistar male rats were used and were submitted to catheterization of the carotid artery and jugular vein to measure the hemodynamic parameters and administrate the extract and drugs, respectively. Thereafter, a dose-administration curve was performed with different doses of the extract, 25, 50, 75, 100, 125, 150, 175, and 200 mg/kg of accumulated extract were administrated in these animals. Blood pressure decreased with the extract, from 50 mg/kg, in a dose-dependent manner and the heart rate was reduced in the presence of the extract, from 50 to 200 mg/kg.

To understand the possible mechanisms of the hypotensive effect by *C. xanthocarpa*, a NO synthesis inhibitor known as *N*-nitro-l-arginine methyl ester (L-NAME) (30 mg/kg) was used to evaluate NO synthase mechanism. Moreover, losartan (10 mg/kg), an angiotensin receptor-1 (AT1R) antagonist of angiotensin II, was administered to evaluate AT1R mechanism through angiotensin II. Hexamethonium (20 mg/kg), a ganglionic blocker, was also used to evaluate the involvement of sympathetic ganglionic blockade mechanism. Thus, the results have demonstrated that the hypotensive action of the extract may be involved in the AT1R mechanism through a sympathetic autonomic response [47].

In this way, angiotensin II may bind to AT1R, promoting the pathophysiological changes on blood pressure, such as abnormal vascular smooth muscle cell contraction, high levels of aldosterone secretion, excessive renal sodium reabsorption, and inappropriate cardiovascular responses [72]. However, previous evidence showed that when AT1R is blockaded, it may result in a reduction of oxidative stress, inflammatory markers, such as pro-inflammatory cytokines, and fibrinolysis inhibition [73]. Together with these findings, phenolic compounds, such as gallic acid, quercetin, and chlorogenic acid, were found during a phytochemical analysis. The high content of quercetin in the *C. xanthocarpa* extract may be primarily responsible for its hypotensive action [47].

Though most of the studies were not necessarily performed on metabolic and pathological conditions in experimental models of obese rodents or in obese human subjects, the studies were accomplished to understand the effects of *C. xanthocarpa* on metabolic dysfunctions that are commonly associated to the obesity profile. Therefore, the results have demonstrated that *C. xanthocarpa*, together with its bioactive compounds, promoted beneficial effects in pathological conditions associated to obesity (Table 1).

### 2.2. Campomanesia adamantium (Cambess.) O. Berg

*C. adamantium* (Cambess.) O. Berg is also found in many regions of Brazil, such as the Cerrado region [22,74]. It is a small tree that produces edible fruits which, due to its well-appreciated taste, are used to produce food products like jellies, juices, and liqueurs. Moreover, this plant has been used in the traditional medicine since previous studies have shown beneficial effects on health from *C. adamantium* fruits, such as antimicrobial [75,76], antidepressant, antihyperalgesic, and anti-inflammatory effects [77], antidiarrheal activity [78], and antiproliferative action [38,79]. In addition to these effects, other studies described that leaves and roots have exhibited antiproliferative action against prostate cancer cells [38] and, more recently, promoted apoptotic death of leukemic cells [39]. It is also said to demonstrate anti-inflammatory and antinociceptive effects [31], antioxidant effects [33,34,35,36,79], and antimicrobial activity [80].

Regarding studies that focus on obesity and its endocrine dysfunctions, there are still few studies that demonstrate the effects of *C. adamantium* on these conditions. An important study performed by Espindola et al. [37] showed that *C. adamantium* root aqueous extract (ExCA) exhibited both antihyperlipidemic and antioxidant effects in hyperlipidemic Wistar rats that received a high-fructose diet (HFD) for 16 weeks. After hyperlipidemia assessment, the animals continued receiving HFD concomitantly with an oral gavage treatment and, therefore, were divided in four treatment groups: HFD (HFD + 300 µL of water), HFD-S (HFD + 30 mg of simvastatin per kilogram of body weight), HFD-C (HFD + 2 mg ciprofibrate per kilogram of body weight), and HFD-ExCA (HFD + 200 mg of *C. adamantium* root aqueous extract per kilogram of body weight). There was also a normolipidemic group (CT) which received a standard rodent commercial chow diet + 300 µL of water, totaling eight weeks of treatment. 

The results have showed that the ExCA group displayed a reduction in serum levels of total cholesterol and triglycerides when compared with the HFD group. Similar findings were observed in the HFD-S, HFD-C, and CT groups. Both simvastatin and ciprofibrate are two drugs that are currently used for the treatment of hypercholesterolemia and hypertriglyceridemia, respectively. In this way, because hypertriglyceridemia is usually accompanied by high levels of cholesterol [81], ExCA was able to reduce both of these conditions. Therefore, the possible mechanism involved in cholesterol reduction is the inhibition of HMG-CoA reductase, as demonstrated by [68] for *C. xanthocarpa*. Regarding other metabolic parameters, ExCA was not able to diminish body weight and organ mass, except liver mass which displayed an increase without changes on the hepatic enzymes alanine aminotransferase (ALT) and aspartate aminotransferase (AST) [37].

Besides these metabolic effects, the chemical profile showed the presence of gallic and ellagic acid and flavonoids which, in turn, led to the analysis of the antioxidant activity of ExCA. First, the 2,2-diphenyl-1-picrylhydrazyl (DPPH) free radical scavenging activity was evaluated to ascertain the in vitro antioxidant activity, demonstrating an efficiency in antioxidant activity with a half-maximal inhibitory concentration (IC_50_) similar to that of butylhydroxytoluene (BHT), a reference antioxidant. To evaluate the antioxidant activity of ExCA in an in vitro cellular model, the extract was demonstrated to be able to protect human erythrocytes, a common in vitro cellular model, against the hemolysis induced by 2,2′-azobis (2-methylpropionamidine) (AAPH) dihydrochloride. Consequently, the lipid peroxidation of erythrocytes induced by AAPH was evaluated through the dosage of malondialdehyde (MDA) in vitro, a product of lipid peroxidation of erythrocyte. In turn, ExCA led to a concentration-dependent reduction in the generation of MDA, similar to ascorbic acid. Thus, these in vitro results were confirmed in vivo, since there was a reduction of MDA in the serum of hyperlipidemic animals treated with ExCA, confirming the antioxidant activity of the extract [37].

It is notable that phenolic compounds are one of the most important classes of secondary metabolites of plants that display the capacity for free radical scavenging [10,82]. Therefore, the main bioactive compounds found in ExCA, such as ellagic and gallic acid [37], display the capacity to capture free radicals and may also stimulate the activity of the antioxidant enzymes, such as superoxide dismutase (SOD), catalase (CAT), and glutathione peroxidase (GPx) [83], together with the attenuation of diabetic cardiomyopathy in mice [84].

Some studies have also evaluated the antioxidant activity of the *C. adamantium* plant. A current study performed by Alves et al. [85] showed that fruits from *C. adamantium* displayed the higher acid ascorbic (vitamin C) content in comparison to the other plants, such as cagaita (*Eugenia dysenterica* DC.) and the cerrado cashew (*Anacardium othanianum* Rizz.). Yet, the antioxidant capacity was assessed through DPPH, ferric reducing antioxidant power (FRAP), and oxygen radical absorption capacity (ORAC) tests which, in turn, demonstrated that even there was significant variation among the plants, *C adamantium* exhibited the highest antioxidant capacity due its high quantity of phenolic compounds, particularly catechin [85].

The antioxidant activity observed from *C. adamantium* fruits corroborates another study that which used air-dried and powered *C. adamantium* leaves collected from four distinct regions of the Brazilian Cerrado during the flowering stage [34]. All extracts have displayed a high antioxidant activity, with a wide range of radical-scavenging (assessed through the DPPH method) and a high inhibition of peroxidation. The latter was assessed through the β-carotene-linoleic acid assay, a test that has been used to mimic the oxidation of the membrane lipid components in the presence of antioxidants in the cells. In addition, the antioxidant capacity is assessed by measuring the inhibition of the organic compounds and the conjugated diene hydroperoxides from linoleic acid oxidation [86,87].

Furthermore, with the purpose of evaluating the bioactive compounds present in the leaves, five flavanones and three chalcones were isolated from *C. adamantium* leaves, being analyzed through high performance liquid chromatography (HPLC). Phenolic content was also evaluated [34]. In general, it was observed that the antioxidant activity, by inhibition of peroxidation, may have a correlation, at least in part, to phenolic content. This is because the production, concentration, and type of secondary metabolites in *C. adamantium*, as well as in other plants in general, may be influenced by the environmental strain, climate, and soil of each region [34].

On the contrary, another study has evaluated the antioxidant activity of *C. adamantium* essential oils through the DPPH method [80]. *C. adamantium* leaves were collected from four distinct cities of Brazilian Cerrado regions. Then, the essential oils were isolated from a 400 g quantity of fresh *C. adamantium* leaves. These essential oils have presented a lower antioxidant activity due to the absence or lower quantity of donor groups of the electron in the ortho position in relation to phenolic hydroxyl. This acted together with larger amounts of hydrocarbons terpenes, which were one of the predominant bioactive compound classes identified. Apart from the evaluation of antioxidant activity, *C. adamantium* leaf essential oils were characterized using gas chromatography-mass spectrometry (GC-MS) in the reproductive (flowering and fruit-bearing) and vegetative stages. This identified a total of 95 compounds, including terpenic hydrocarbons, ether, alcohol, aldehydes, ketones, esters, phenols, and epoxides. In the reproductive stage the main bioactive compounds found were monoterpenes, such as limonene, α-pinene, and β-pinene; whereas, in the vegetative stage, the major constituents were the sesquiterpenes, bicyclogermacrene, and globulol [80].

Although further studies are necessary to investigate the potential of *C. adamantium* on pathological conditions associated with obesity, in vivo studies are still necessary to evaluate the bioavailability of these bioactive compounds in rodent models and especially in the human biological system (Table 2), concomitant with the evaluation of metabolic parameters associated to obesity and the bioactive compound′s mechanisms of action.

### 2.3. Campomanesia pubescens O. Berg

*C. pubescens* is another member of the *Campomanesia* genus and is normally found in the southeast and middle west of Brazil. In the same way as *C. xanthocarpa* and *C. adamantium*, this variety is known due to its medicinal properties [75,87,88], and its fruits display a fleshy pulp. When the *C. pubescens* fruits are ripe, they exhibit a high vitamin C content (1000 mg/100 g) and phenolic compounds [89]. As demonstrated in different species of Brazilian Cerrado *Campomanesia* species, they are sources of bioactive compounds that present high antioxidant activity, indicating their therapeutic potential in the prevention and treatment of some diseases [88].

Even though the studies directed to *C. pubescens* are still scarce, previous studies have showed that the essential oils extracted from different parts of *C. pubescens* possess different bioactive components. The leaves have demonstrated a high concentration of phenolic and proanthocyanidins, such as sesquiterpenes like germacrene-D, eucalyptol, and trans-sabinene hydrate. The essential oil of fruits displayed higher concentrations of monoterpene, especially limonene, eucalyptol, α-pinene, and α-terpineol. In addition to the leaf and fruit essential oils, the major bioactive components found in stem essential oils were eucalyptol, spathulenol, bicyclogermacrene, and germacrene-D [90] Another study showed that *C. pubescens* contained 34 volatile compounds, such as α-pinene and β-caryophyllene, together with flavanones [76].

*C. pubescens* has showed antioxidant activity. Cardoso et al. [88] found that the hexanic extract from *C. pubescens* leaves had sesquiterpenes and triterpenes, which have low polarity and show a high antioxidant activity. However, the samples have low antioxidant activity, as detected by the DPPH method (which reacts with polar samples), and have high antioxidant activity, as detected by β-caroten/linoleic acid method (which reacts with non-polar samples). This may have occurred because of the substances sequestrant of oxygen as a function of the double bonds present in its structure. These were then assigned to the identification of antioxidant activity by the second method. On the other hand, the antioxidant activity from the ethanolic extracts from different parts of *C. pubescens* was also demonstrated, through the DPPH method, due to phenolic compounds contents [89].

Another work showed that two types of extracts from *C. pubescens* fruits, including hexanic and ethyl acetate extracts, have presented chalcones such as 2′,4′-dihydroxy-3′,5′- dimethyl-6′-methoxychalcone and 2′,4′-dihydorxy-5′-methyl-6′-methyl-6′methoxychalcone. Comparing the antioxidant activity of both extracts through the DPPH method, the ethyl acetate extract has displayed a higher antioxidant activity than the hexanic extract; however, the last extract obtained the highest quantity of total phenolic compounds. Yet, both extracts have exhibited antitumoral activity in the human tumor lineage in vitro assay of melanoma (UACC-62), breast (MCF-7), prostate (PC-3), and colon (HT-29) cancers [91].

In order to evaluate the toxicity effects from *C. pubescens*, Guerrero et al. [92] observed the effects of the hydroethanolic extract from *C. pubescens* leaves of different concentrations (250 and 500 mg/kg), administered through oral gavage, for 90 days in rats. It was able to reduce the number of monocytes when compared to the control group, which displayed a superior number of monocytes according to the reference values, suggesting that this extract may present an anti-inflammatory potential. Moreover, the control group showed increased levels of ALT and AST, unlike the treated group which demonstrated decreased plasma levels of these hepatic enzymes without renal toxicity.

It is notorious that studies related to the *C. pubescens* variety, especially regarding metabolic dysfunctions associated to obesity, are still scarce. However, the works that were already accomplished may be key points to be carried out in models of obesity-associated metabolic syndrome, since *C. pubescens* demonstrated anti-inflammatory and antioxidant properties (Table 3). Thus, further research that highlights the possible therapeutic actions of this plant, especially in metabolic disorders, may be performed.

## 3. Conclusions

This review evaluated the effect of treatment with different doses of species from the *Campomanesia* genus, belonging to the family Myrtaceae. In turn, these studies have demonstrated that this plant displays effects on health, such as antiulcerogenic, antiprotozoal, anti-inflammatory, antioxidant, and antiproliferative activities in animal models, human clinical trials, and also in in vitro assays. *C. xanthocarpa* was extensively evaluated in comparison to the other *Campomanesia* species. Compounds of interest included *C. xanthocarpa*, which was associated to anti-inflammatory and antinociceptive effects, as well as hypoglycemic activity and antiplatelet activity. *C. adamantium* demonstrated antioxidant activity and hypolipidemic effects, lowering both cholesterol and triglycerides. *C. pubescenses* presented with antitumoral activity in human tumor lineage in in vitro assays of melanoma, breast, prostate, and colon cancers, along with antioxidant capacity. Regarding the concern of metabolic disturbances associated to obesity, the use of *Campomanesia* species in the treatment of metabolic disorders is still under investigation. Therefore, more compounds of interest in metabolic studies may be found, with the *Campomanesia* species being of great potential for the health strategy. Nonetheless, more studies are required to standardize safe concentrations, assess possible side effects, and determine mechanism of actions, especially in obesity-induced metabolic syndrome in animal models and human clinical trials.

## Figures and Tables

**Table 1:** Principal effects of *C. xanthocarpa* in metabolic disturbances.

Host	Effects	Extract/Doses	Reference
Rats	Reduced weight gain Decreased blood glucose levels	Leaf extract (infusion ad libitum)	[49]
	Decreased blood glucose levels Inhibited hepatic glycogen loss Preserved histopathological alterations in the pancreas without glomerular alterations	Leaf decoction (20g/L)	[50]
Mice	Demonstrated antiplatelet, antithrombotic without cytotoxic effects and gastric lesions	Leaf extract (30 and 100 mg/kg/day) and ASA (100 mg/kg/day)	[45]
	Attenuated proinflammatory markers, such as IL-6, IL-1, TNF-α, and IFN-γ Increased IL-10 Only *C. xanthocarpa* was able to decrease anti-oxLDL antibodies without ulcerogenic activity	Leaf extract (100 mg/kg/day) and ASA (100 mg/kg/day)	[58]
Rats	Decreased blood pressure in a dose-dependent manner (50 mg/kg) Reduced heart rate (50 to 200 mg/kg)	Leaf extract (25, 50, 75, 100, 125, 150, 175, and 200 mg/kg)	[47]
Humans	Decreased LDL-c and total cholesterol levels without differences in triglyceride, VLDL-c, or HDL-c levels Reduced inflammation and oxidative stress Displayed protective effects on the endothelium.	Encapsulated leaf extract (500 mg, 750 mg or 1000 mg)	[42]
	Demonstrated antiplatelet effects	Leaf extract (1000 mg), ASA (100 mg), ASA (50 mg) + leaf extract (500 mg)	[46]

Principal studies of *C. xanthocarpa* in metabolic disorders accomplished in animal models (in vivo) and human clinical trials.

**Table 2:** Possible effects of *C. adamantium* in metabolic disturbances.

Host	Effects	Extract/Doses	Reference
Antioxidant activity assays	Exhibited high antioxidant activity and high capacity to inhibit peroxidation	*C. adamantium* leaves extract (different regions of Brazilian Cerrado)	[34]
	Exhibited low antioxidant activity	*C. adamantium* leaves essential oils (different regions of Brazilian Cerrado)	[80]
	High antioxidant activity	Fruits from *C. adamantium*	[85]
Rats and In vitro	Exhibited antihyperlipidemic and antioxidant effects High antioxidant activity (antioxidant activity assay)	Root extract (200 mg/kg)	[37]

Principal studies of *C. adamantium* with possible effects in metabolic disorders accomplished in animal models (in vivo) and to evaluate its antioxidant activity.

**Table 3:** Possible effects of *C. pubescens* in metabolic disturbances.

Host	Effects	Extract/Doses	Reference
Antioxidant activity assay	Exhibited high antioxidant activity	Leaf extract	[88]
		Root, stem, leaf and fruit extracts	[89]
Antioxidant activity assay and In vitro	Exhibited high antioxidant activity together with antitumoral activity in the human tumor lineage in vitro assay	Fruit extracts	[91]
Rats	Reduced the number of monocytes (anti-inflammatory potential) Decreased plasma levels of hepatic enzymes (ALT and AST) withour renal toxicity	Leaf extract (250 and 500 mg/kg)	[92]

Principal studies of *C. pubescens* with possible effects in metabolic disorders accomplished in animal models (in vivo) and to evaluate its antioxidant activity.
